# Inhibition of Sunn Pest, *Eurygaster integriceps,* α-Amylases by α-Amylase Inhibitors (T-αAI) from Triticale

**DOI:** 10.1673/031.010.14139

**Published:** 2010-10-13

**Authors:** Mohammad Mehrabadi, Ali R. Bandani, Fatemeh Saadati

**Affiliations:** Insect Biochemistry & Molecular Biology Lab, Plant Protection Dep., Agricultural and Natural Resources Campus, University of Tehran, Karaj, Iran

**Keywords:** Scutelleridae, mode of inhibition

## Abstract

The effect of triticale α-amylases inhibitors on starch hydrolysis catalyzed by the Sunn pest, *Eurygaster integriceps* Puton (Hemiptera: Scutelleridae) midgut amylases was examined. Biochemical studgawies showed that inhibitors from Triticale (a hybrid of wheat and rye) had inhibitiory effects on *E. integriceps* α-amylases. The effects of the triticale α-amylase inhibitor (T-αAI) on α-amylase of *E. integriceps* showed a dose dependent manner of inhibition, e.g. less inhibition of enzyme activity (around 10%) with a lower dose (0.25 mg protein) and high inhibition of enzyme activity (around 80%) when a high dose of inhibitor was used (1.5 mg protein). The enzyme kinetic studies using Michaelis-Menten and Lineweaver-Burk equations showed the K_m_ remained constant (0.58%) but the maximum velocity (V_max_) decreased in the presence of a crude extract of Triticale inhibitors, indicating mixed inhibition. The temperature giving 50% inactivation of enzyme (T_50_) during a 30-min incubation at pH 7.0 was 73° C. The maximum inhibitory activity was achieved at 35° C and pH 5.0. Gel assays showed the meaningful inhibition of *E. integriceps* α-amylases by various concentrations of Triticale inhibitors. Based on the data presented in this study, it could be said that the T-αAI has good inhibitory activity on *E. integriceps* gut α-amylase.

## Introduction

The Sunn pest, *Eurygaster integriceps* Puton (Hemiptera: Scutelleridae), is one of the most serious pests of wheat and barley in the wide area of the Near and Middle East, West Asia, and many of the newly independent states of central Asia. It also is found in Eastern and Southern Europe and North Africa (Rajabi 2000). Yield loss because of *E. integriceps* infestation in some areas is 100%, and because of severe infestation by this insect, many wheat fields are not harvested. *E. integriceps* causes severe quantitative and qualitative damage to crops by feeding on leaves, stems, and grains.

Their feeding is typical of Heteroptera:, piercing and cutting tissues with their stylets while injecting digestive enzymes, amylases, and proteases through their salivary canals to liquefy food into nutrient-rich slurry. The food slurry is ingested through the food canal and passed into the alimentary canal where it is further digested and absorbed (Cohen 2000; Boyd et al. 2002). *E. integriceps* feed on different stages of developing grains. They suck the milky nutrients from the immature grain by piercing it with their mouthparts and injecting saliva that contains very potent enzymes that degrade gluten proteins. Flour from such grain causes rapid relaxation of dough resulting in the production of bread with poor volume and texture (Radjabi 2000).

Many insects, including *E. integriceps,* that constitute serious pests of wheat grain live on a polysaccharide-rich diet and are dependent on their α-amylases for survival (Mendola-Olaya et al. 2000; Boyd et al. 2002). They convert starch to maltose, which is then hydrolyzed to glucose by α-glucosidase. In insects, only α-amylases that hydrolyse α-1,4-glucan chains such as starch or glycogen have been found (Terra et al. 1999).

*E. integriceps* utilize α-amylases for carbohydrate metabolism, and due to the importance of α-amylases for carbohydrate metabolism, different forms of α-amylases have been found in this insect that apparently guarantee effective digestion (Kazzazi et al. 2005; Mehrabadi et al. 2009). Due to its dependence on α-amylases for survival, these enzymes can be good target candidates for bio-insecticides via α-amylase inhibitors (Franco et al. 2002; Svensson et al. 2003; Sivakumar et al. 2006.).

Triticale (X Triticosecale Wittmack) is the product of an artificial cross between wheat (*Triticum aestivum*) and rye (*Secale cereale*) genomes resulting in hexaploid (AABBRR) or octaploid (AABBDDRR) triticales. However, less attention has been given to α-amylase inhibitors of triticale even though they have been isolated from parental wheat and rye (Hernández et al. 1999).

Six different α-amylase inhibitor classes — lectin-like, knottin-like, cereal-type, Kunitz-like, c-purothionin-like, and thaumatin-like — could be used in pest control (Franco et al. 2002). These inhibitors show structural diversity, thus leading to different modes of inhibition and different specificity profiles against a diverse range of α-amylases. Specificity of inhibition is an important issue as the introduced inhibitor must not adversely affect the plant's own α-amylases or the nutritional value of the crop (Franco et al. 2002).

α-Amylase inhibitors are extensively found in many plant seeds and tubers, being particularly abundant in cereals and legumes (Franco et al. 2002; Svensson et al. 2003; Payan 2003; Sivakumar et al. 2006). These molecules play a key role in plant defense toward pests and pathogens that cause severe damage to field crops and stored grains (Koiwa et al. 1997; Franco et al. 2000, 2002; Payan 2003; Svensson et al. 2003). Since these inhibitors could show different specificities against α-amylases from different sources, inhibitors with a wide specificity spectrum are strongly favored for insect control (Franco et al. 2002).

Since large scale pesticide utilization caused deleterious effects to human health and the environment, enzyme inhibitors could be an alternative strategy for control of phytophagous and storage seed insect-pests (Gatehouse and Gatehouse 1998; Franco et al. 2002). Several studies demonstrated the efficiency of enzyme inhibitors against important economic pests from different orders (Leple et al. 1995; Xu et al. 1996; Gatehouse and Gatehouse 1998; Franco et al. 2000, 2002; Sadasivam and Thayumanavan 2003). The aim of the current study was to investigate the inhibitory effects of triticale against *E. integriceps* α-amylase using spectrophotometry and gel assay. Also, the mode of action of the Triticale inhibitors toward *E. integriceps* amylases were explored through kinetic studies using Michaelis— Menten and the derived Lineweaver—Burk equations.

## Materials and Methods

### Insects

One population of *E. integriceps* was collected from a wheat farm during the summer in Karaj, Tehran province in Iran. They were fed and maintained on wheat grains under laboratory conditions at 25 ± 2° C and a photoperiod of 14:10 L:D.

### Exraction of Triticale α-amylase Inhibitor (T-αAI)

T-αAI from seeds of Triticale was extracted according to Baker (1987) and Melo et al. (1999). Ground seeds (30 g each) were mixed with a solution of 0.1*M* NaCl and stirred for two h, followed by centrifugation at 10,000 g for 30 min. The pellet was discarded, and the supernatant was incubated at 70° C for 20 min to inactivate major endogenous enzymes. Fractionation of the supernatant was done using different concentrations of ammonium sulfate (20, 40, 60, and 80%) followed by centrifugation at 10,000 g for 20 min at 4° C. The 60% pellet containing the highest fraction of amylase inhibitors was dissolved in icecold sodium phosphate buffer (0.02 *M* and pH 7.0) and dialyzed overnight against the same buffer. This dialyzed solution was used as a source of amylase inhibitors in enzyme assays.

### Enzyme preparation

Enzyme samples from the midguts of adults were prepared. Adults were randomly selected, and midguts from these individuals were removed by dissection under a light microscope in ice-cold saline buffer (0.006 *M* NaCl). The midgut was separated from the insect body, rinsed in ice-cold saline buffer, placed in a pre-cooled homogenizer, and ground in 1 ml of universal buffer containing succinate, glycine, 2-morpholinoethanesulfonic acid at pH 6.5. The homogenates from both preparations were separately transferred to 1.5 ml centrifuge tubes and centrifuged at 15,000 g for 20 min at 4° C. The supernatants were pooled and stored at -20° C for subsequent analyses.

### Amylase assay

The α-amylase activity was assayed by the dinitrosalicylic acid (DNS) procedure (Bernfeld 1955), using 1% soluble starch (product number 1257, Merck Group, www.merck.de/) as substrate (Bandani et al. 2009). One unit of α-amylase activity was defined as the amount of enzyme required to produce 1 mg maltose in 30 min at 35° C. A standard curve of absorbance against amount of maltose released (Product Number 105911, Merck, Mr 360.32 mg mol^-1^) was constructed to enable calculation of the amount of maltose released during α-amylase assays. All assays were performed in duplicate, and each assay was repeated at least three times.

### Amylase inhibition assay

To determine if the enzyme activity could be inhibited by T-αAI, the assay was conducted using a whole insect midgut extract for amylase activity. Since the *E. integriceps* gut contains most of the α-amylase (Mehrabadi et al. 2009; Bandani et al. 2009) a midgut extract was used. In the inhibition assay, enzyme extract was pre-incubated with different concentrations of T-αAI for 30 min at 30° C; then the same procedure for the amylase assay was conducted, and amylase activity was determined by measuring absorbance at 540 nm.

### Effect of pH on the inhibitory activity of T-αAI

The extent to which T-αAI inhibits *E. integriceps* α-amylase was determined at different pH values using universal buffer (Hosseinkhani and Nemat-Gorgani 2003) with pH set at 2, 3, 4, 5, 6, 7, 8, 9, and 10. Universal buffer consisted of glycine (0.02 *M*), succinate (0.02 *M*) and Mes (2-[morpholino]ethansulphonic acid) (0.02 *M*). Adjustment of pH was done by addition of NaOH (1.0 N). The amylase activity remaining after 30 min incubation at 30° C and in the presence of T-αAI (1.5 mg/ml) was determined by adding 0.4 ml of 1% starch solution. Controls were run at each pH value with midgut amylase alone as a control, and the percentages of inhibition were calculated from the controls vs. inhibited midgut amylase values measured at each pH.

### Time- and temperature-dependence of *E. integriceps* α-amylase Inhibition

Time course inhibition *E. integriceps* midgut α-amylase by T-αAI carried out by pre-incubation of enzyme extract with T-αAI in 2 m*M* sodium phosphate buffer (pH 5.0) at 25, 30, and 35° C for different times. To determine the time dependence of the thermal inactivation of the inhibitor, T-αAI was incubated in 2 m*M* sodium phosphate buffer at 50, 60, 70, and 80, ° C for 10, 20, 30, 40, 50, and 60 min, and then the remaining amylase inhibitory activity was assessed.

### Kinetic of inhibition

The inhibition was measured with increasing concentrations of starch as a substrate (0.5– 2.0%) in the presence of different concentrations (0.2 to 1.5 mg/ml protein) of T-αAI. The type of inhibition was determined by Lineweaver-Burk plot analysis of the data, which was calculated from the results according to Michaelis-Menten kinetics. The inhibitory constants (K_i_) and dissociation constants of enzyme-substrate inhibitor complexes (K'_i_) were determined (Dixon 1953; Bowden 1974). All the experiments were repeated three to four times.

### In gel inhibitory assay of amylase

Enzyme extract was pre-incubated with different concentrations of T-αAI for 30 min at 30° C, and then the remaining amylase activity was determined by SDS-polyacrylamide gel electrophoresis (Native Page). SDS-PAGE was carried out using the procedures described by Lammli (1970) and Campos et al. (1989), which were modified for *E. integriceps.* SDS-PAGE was performed in 10% (w/v) gel with 0.05% SDS for separating gel and 5% for stacking gel with 0.05% SDS.

The electrode buffer was prepared based on the method of (Lammli 1970), but SDS was not used. The sample buffer contained 25% stacking buffer (0.5 *M* Tris-Hcl, pH 6.8), 20% glycerol, 2% SDS, 0.005% (w/v) bromophenol blue, but without mercaptoethanol or heating. Electrophoresis was conducted at a voltage of 120V until the blue dye reached the bottom of the slab gel. To prepare gels for α-amylase assay, the gel was rinsed with distilled water and washed by shaking gently with 1% (v/v) Triton X-100 in phosphate buffer containing 2 mM CaCl_2_ and 10 mM NaCl for 30 min. Then, the gel was rinsed with distilled water and treated with a 1% starch solution for 1.5 h. Finally, after rinsing with distilled water, the gel treated with a solution of 1.3% 12, 3% KI to stop the reaction and to stain the un-reacted starch background. Zones of α-amylase activities appeared as a light band against the dark background of the gel.

**Figure 1.**
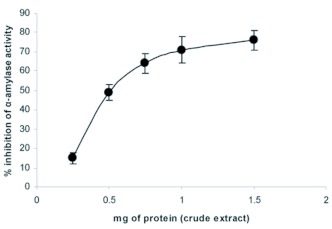
Inhibition of *Eurygaster integriceps* α-amylase by crude inhibitor preparation of Tritcale. Enzyme and inhibitor were incubated for 30 min prior to the addition of starch to measure enzyme activity. Each point represents the average of three measurements. High quality figures are available online.

### Protein determination

Protein concentration was measured according to the method of Bradford (1976), using bovine serum albumin (Bio-Rad, www.bio-rad.com) as standard.

## Results and Discussion

### Effects of T-αAI on *E. integriceps* amylase activity

The effects of T-αAI on α-amylase of *E. integriceps* showed a dose dependent manner of inhibition e.g. the lower dose (0.25 mg) of inhibitor around 10% enzyme inhibition was achieved while the highest dose (1.5 mg) of inhibitor caused an 80% inhibition of amylase activity (Figure 1). These results are in agreement with other reports; for example, Valencia et al. (2000) reported that at 1 mg/ml of crude protein of *Amaranthus cruentus* protein caused an 80% inhibition of coffee berry borer, *Hypothenemus hampei,* amylase activity, whereas the *Amaranthus* hybrid crude protein inhibited only 40%. Furthermore, they showed addition of 1 mg of the bean seed protein (crude inhibitor) caused an 80% inhibition of amylase activity. Also, Melo et al. (1999) reported that α-amylase inhibitor extracted from cowpea seeds, *Vigna unguiculata,* inhibited α-amylase from *Callosobruchus maculates* larvae by 50%.

Since biochemical properties of *E. integriceps* salivary amylase appear similar to gut amylases (Kazzazi et al. 2005), the results obtained with midgut extract may also reflect salivary α-amylase activity, but further experiments are required to test this assumption.

### pH dependence inhibition activity of T-αAI

**Figure 2.**
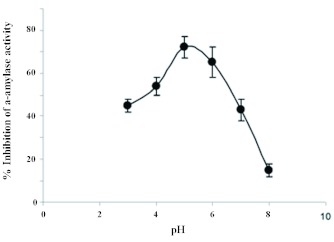
pH dependence of the inhibitor activity of Triticale α-amylase inhibitor towards *Eurygaster integriceps* α-amylase. *E.* *integriceps* midgut extract was incubated with Triticale α-amylase inhibitor (1.5 mg/ml) at various pHs. Each point represents the average of three measurements. High quality figures are available online.

The extent of the inhibition of *E. integriceps* α-amylase by T-αAI was dependent on the pH value of the assay medium (Figure 2). As can be seen the highest inhibitory effect was seen at pH 5.0. The inhibition dropped markedly outside the narrow optimum pH of 5.0 at 30° C. Between pH 3.0, 5.0, and 8.0 α-amylase was inhibited by more than 40% (Figure 2).

It has been reported that the interaction between amylase and amylase inhibitor is pH-dependent, with an optimum around pH 4–5 (Marshall and Lauda 1975; Powers and Whitaker 1977; Valencia et al. 2000). For example, there is an optimum pH of 5.5 for inhibition of porcine pancreatic alpha-amylase (PPA) by kidney bean, *Phaseolus vulgaris* αAI varies between 4.5 to 5.5 depending on the strain used (Marshall and Lauda 1975; Powers and Whitaker 1977; Lajolo and Finardi-Filho 1985), and an optimum pH of 5.0 for inhibition of coffee berry borer (*H. hampei*) amylase inhibitor (αAI-1) from the common bean, *P. vulgaris and Amaranthus* (Valencia et al. 2000).

The insect gut lumen is the place where the interaction between α-amylase and T-αAI occurs. It has been reported that *E. integriceps* has an acidic midgut pH range of 5.5–6.5, and α-amylase has a slightly acidic optimum pH (6.5) (Mehrabadi et al. 2009). Therefore, it can be expected that α-amylase activity would be high under such conditions and for it to be inhibited if T-αAI is present at a high enough concentration. The accordance between gut lumen pH, amylase optimal pH, and pH optimum for amylase inhibition by plant amylase inhibitors has been described in other insect studies (Biggs and McGregor 1996; Valencia et al. 2000).

### Thermal stability and time course inhibition of *E. integriceps* amylase by T-αAI

**Figure 3.**
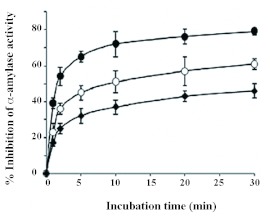
Time course inhibition of *Eurygaster integriceps* α-amylases by Triticale α-amylase inhibitors. *E. integriceps* midgut extract was pre-incubated with Triticale α-amylase inhibitor in 2 m*M* sodium phosphate buffer (pH: 5.0) at 25° C (diamond), 30° C (open circle), and 35° C (closed circle). Each point represents the average of three measurements. High quality figures are available online.

The data showed that the maximum α-amylase inhibition by T-αAI was achieved after 20–30 min of incubation (Figure 3). This result is in accordance with that of Marshall and Lauda (1975) who reported 60–70% inhibition of PPA by *P. vulgaris* amylase inhibitor at 37° C and LeBerre-Anton et al. (1997) who observed maximum inhibitory activity of α-amylase inhibitor from *P. vulgaris* seeds toward PPA after 10 min.

The effect of three different temperatures on the inhibition of *E. integriceps* α-amylase by T-αAI was recorded at the optimum pH of 5.0. The inhibition percentages obtained at 25, 30, and 35° C were 42, 55, and 81%, respectively showing that increasing temperature increases inhibitory activity of T-αAI. Marshall and Lauda (1975) reported a 10-fold increase in activity of the α-amylase inhibitor when the temperature of the reaction was raised from 25 to 37° C. LeBerre-Anton et al. (1997) reported that temperature had a moderate effect on the activity of α-All. Interestingly, Mehrabadi et al. (2009) observed maximum *E. integriceps* α-amylase activity at a range of 30–35° C which revealed that *E. integriceps* α-amylase and T-αAI have the maximum activities within the same temperature range.

**Figure 4.**
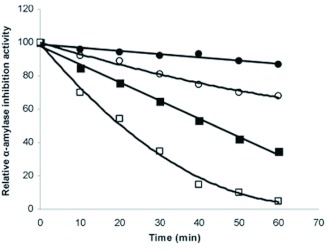
Time dependence of the thermal inactivation of Triticale α-amylase inhibitor. Triticale α-amylase inhibitors was incubated in 2 m*M* sodium phosphate buffer at 50 (closed circles), 60 (open triangles), 70 (close squares), and 80° C (open squares), for indicated time. Each point represents the average of three measurements. High quality figures are available online.

Thermal inactivation of T-αAI was observed at pH 5.0 in the temperature range of 50–80° C (Figure 4). *E. integriceps* α-amylase activity was measured at 30° C in the presence of the heat-treated T-αAI. It was found that increasing heat treatment time of T-αAI caused inactivation of T-αAI. The temperature giving 50% inactivation (T_50_) in a 30-min incubation time was determined to be 73° C. Oneda et al. (2004) reported that T_50_ of α-amylase inhibitor from wheat kernel on the activity of porcine pancreas α-amylase (PPA) was 88° C in 30-min incubation time at pH 6.9.

### Mode of inhibition of *E. integriceps* α*-*amylase by T-αAI

**Figure 5.**
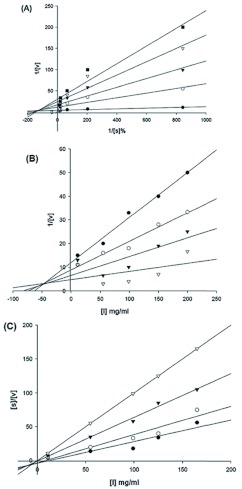
Lineweaver—Burk and Dixon plots of *Eurygaster integriceps* α-amylase in the presence of Triticale α-amylase inhibitors, which provide an estimation of *K_i_* (the inhibition constant or the dissociation constant of EI). A: Lineweaver-Burk plot of the amylase hydrolysis reaction with variable starch concentrations (0.25–1.5%) and at fixed concentration of crude Triticale α-amylase inhibitors, i.e., 0 (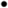), 0.25 (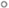), 0.5 (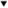), 1 (Δ), and 1.5 mg/ml (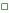). B: Dixon plot of the amylase hydrolysis reaction with variable concentrations of Triticale α-amylase inhibitors at a fixed substrate concentration as follows: 0.005% (Δ), 0.2%(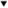), 0.3%(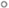)and 0.6%(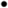). C: the plot of *slv* against *i*, which provides complimentary information for distinguishing inhibition types (the dissociation constant of the EIS complex). High quality figures are available online.

To determine whether inhibition of α-amylase by T-αAI was competitive or non-competitive, Michaelis-Menten and Lineweaver-Burk plots were drawn for the non-inhibited and partly inhibited enzyme. In the presence of T-αAI, the slope of the straight lines in a double reciprocal plot increased with increasing concentrations of T-αAI. The straight lines were intercepted at a single point in the second quadrant indicating mixed non-competitive inhibition (Figure 5A). The Dixon plot of the amylase hydrolysis reaction with variable concentrations of Triticale α-amylase inhibitors at a fixed substrate concentration exhibited different slopes and intersected at different locations in the third quadrant. The crude extract had an inhibitory constant, K*i*, value of 45.5 mg (Figure 3B). When *s/v* against *i*, which provides complimentary information for distinguishing inhibition types, was plotted, intercepts occurred in the third quadrant, suggesting mixed non-competitive inhibition (Figure 3C).

A non-competitive inhibitor binds to an inhibitor site on the enzyme that is remote from the active site and brings about a conformational change in the active site. In this sense, it is very similar to one of the competitive inhibitor types. The difference is that the change in the active site is such that it does not prevent substrate binding but, rather, prevents the enzyme from converting the bound substrate to product. A classical noncompetitive inhibitor has absolutely no effect on substrate binding. In fact, a change to the shape of the active site is almost certain to alter the ability of the substrate to bind. It won't stop it altogether, but the affinity will be reduced (Dixon 1953). Inhibitors like this are often called mixed inhibitors, as they appear to have some of the properties of competitive and noncompetitive types. The non-competitive inhibition exerted by T-αAI on *E. integriceps* α-amylase showed T-αAI can bind to the E or to the ES complex other than the catalytic site. The kinetic data indicated that two complexes could be produced: the amylase/T-αAI complex (EI), resulting from binding of the T-αAI compound to the active site, and the amylase/starch-T-αAI complex (ESI) in which T-αAI is bound at a secondary binding site other than the active centre, which is accessible only after starch binding has occurred at the active centre. This treatment assumes that enzyme-inhibitor interaction is not irreversible.

Mixed noncompetitive inhibition has been reported for other amylase inhibitors, i.e., inhibition of PPA by α-amylase inhibitor from *P. vulgaris* seeds (LeBerre-Anton et al. 1997) and inhibition of finger millet malt amylases by the millet phenolics (Chethan et al. 2008). However, Marshall and Lauda (1975) reported a non-competitive inhibition for α-AI from *P. vulgaris* var. Great Northern against porcine pancreatic α-amylase (PPA).

### In gel inhibition assay

**Figure 6.**
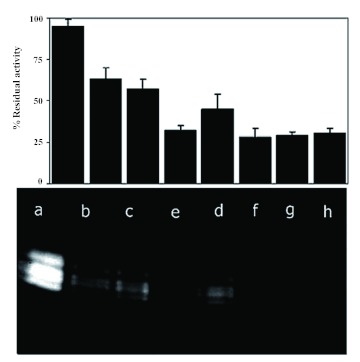
*Eurygaster integriceps* gut extract zymogram using 1% starch as substrate. Enzyme extract was pre-incubated with different concentrations of T-αAI for 30 min at 30 °C, then, and the remaining amylase activity was determined by measuring absorbance at 540 nm and SDS-polyacrylamide gel electrophoresis. SDS-PAGE was performed in 12% (w/v) gel with 0.05% SDS for separating gel and 5% for stacking gel with.0.05% SDS. The sample buffer contained 25% stacking buffer (0.5 M Tris-HCl, pH 6.8), 20% glycerol, 2% SDS, and 0.005% (w/v) bromophenol blue. The gel was stained with a solution of 1.3% 12, 3% Kl. (a) crude extract midgut with no inhibitor, (b) 0.15 mg·ml^-1^ T-αAI, (c) 0.35 mg·ml^-1^ T-αAI, (d) 0.75 mg·ml^-1^ T-αAI, and (e-h) 1.5 mg·ml^-1^ T-αAI, respectively. High quality figures are available online.

Since colorimetric assay showed amylase activities were inactivated by T-αAI, it was of interest to see if the inactivation occurred due to the decomposition of the enzyme complex. The enzyme was subjected to a series of non-denaturing PAGE after the incubation of enzyme extract with different concentrations of T-αAI (Figure 6). Although about 70–80% amylase inhibition was achieved, no significant change of mobility or band intensity was detected in non-denaturing-PAGE (Figure 6) during the incubation time or by inhibitor concentration. This result suggests that the functional inactivity of *E. integriceps* amylases on T-αAI treatment was not due to the dissociation of the enzyme complex.

It has been reported that insect-pests have more than one α-amylase isozymes excreted by digestive tissues. The presence of a number of α-amylases isozymes is a strategy to escape from inhibitor toxicity (Silva et al. 1999). Production of several isozymes was detected for *Sitophilus zeamais,* *Callosobruchus maculatus,* Zabrotes *subfasciatus* and *Acanthoscelides obtectus* (Silva et al. 1999; Franco et al. 2005). In *E. integriceps,* five major isozymes were detected by electrophoresis and were present in all treatments, but their activities were lower compared with the control (Figure 6). The results indicated that the inhibition activity of T-αAI was effective on all detected isozymes, but this inhibition activity did not lead to complete deletion of isozymes. Valencia et al. (2000) showed that inhibitor from *P. vulgaris* cv. Greensleeves effectively inhibited *H. hampei* amylase activity when the gel was incubated for 1 h at 30° C with 5 mg/ml of crude inhibitor. Sivakumar et al. (2006) reported gel inhibition of α-amylase from *Sitophilus oryzae, Tribolium castaneum, Callosobruchus chinensis*, *Carcyra cephalonica, Spodoptera litura, Helicoverpa armigera, Acaea Janata,* and *Plutela xylostella* by little and finger millet inhibitors, which were pre-incubated with midgut crude extract for 60 min at 30° C.

Due to existence of more than one α-amylase isozyme in *E. integriceps,* the specificity of the inhibitor is an important primary step in developing molecules that could be used for production of insect resistant transgenic plants. An efficient inhibitor should have two important properties: (a) it should inhibit the insect enzyme substantially at a low enough concentration and at the pH found in the insect gut and (b) it should be resistant to attack by insect gut proteases (Valencia et al. 2000; Morton et al. 2000).

Based on the data presented here, it could be said that T-αAI has a strong inhibitory activity on *E. integriceps* gut α-amylase. However, the present study is the first study of this kind, so more information, such as on the effect of gut protease on T-αAI, should be obtained.

## Abbreviations

PPA,: porcine pancreas α-amylase;
T-αAI,: triticale α-amylase inhibitor;
K_i_,: Inhibitory (dissociation) constant for the enxyme-inhibitor complex;
K'_i_,: dissociation constant for inhibitor from enzyme-substrate-inhibitor complex;
T50,: The temperature giving 50% inactivation
